# Uncovering therapeutic opportunities in the clinical development of antibody‐drug conjugates

**DOI:** 10.1002/ctm2.1329

**Published:** 2023-09-22

**Authors:** Cristina Nieto‐Jiménez, Adrián Sanvicente, Cristina Díaz‐Tejeiro, Víctor Moreno, Alfonso lopez de Sá, Emiliano Calvo, Joaquín Martínez‐López, Pedro Pérez‐Segura, Alberto Ocaña

**Affiliations:** ^1^ Experimental Therapeutics Unit Hospital Clínico San Carlos (HCSC) Instituto de investigación sanitaria San Carlos (IdISSC) Madrid Spain; ^2^ Facultad Ciencias Químicas Universidad Complutense Madrid Spain; ^3^ START Madrid‐FJD Hospital Fundación Jiménez Díaz Madrid Spain; ^4^ Hospital Universitario 12 de Octubre‐Centro Nacional de Investigaciones Oncológicas (H12O‐CNIO) Haematological Malignancies Clinical Research Unit Spanish National Cancer Research Centre Madrid Spain; ^5^ Department of Hematology, Hospital Universitario 12 de Octubre‐Universidad Complutense Instituto de Investigación Sanitaria Hospital 12 de Octubre (imas12) Madrid Spain; ^6^ Breast cancer Centro de Investigación Biomédica en Red en Oncología (CIBERONC) Madrid Spain

**Keywords:** ADC, clinical approach, new therapies, targeted therapy

## Abstract

**Introduction:**

Antibody‐drug conjugates (ADCs) are a family of therapeutic agents that have demonstrated clinical activity in several indications.

**Material and methods:**

In this article, we performed a deep analysis of their clinical landscape matched with public genomic human datasets from tumour antigen targets (TATs), to identify empty areas for clinical development.

**Results:**

We observed that TATs used in haematological malignancies were more specific than the ones developed in solid cancers. Those included CD19, CD22, CD30, CD33 and CD79b. In solid tumours, we identified TATs, with approved ADCs, widely expressed in non‐explored niche indications like Enfortumab vedotin (anti‐Nectin4) in lung or cervical cancer; Tisotumab vedotin (anti‐TF) in glioblastoma or pancreatic cancer; and Sacituzumab govitecan (anti‐TROP2) in pancreatic, gastric, thyroid or endometrial cancer, among others. Similarly, niche indications for ADCs in clinical development included targets for CD71, PSMA, PTK7 or CD74, in tumours like breast, lung, stomach or colon. Some of these TATs were essential for the survival of tumour cells like CD71, PSMA and PTK7.

**Conclusions:**

In summary, our study opens the door for further evaluation of ADCs in several indications not explored before.

## Graphical abstract

• Most ongoing Antibody‐drug conjugates (ADCs) have a classical structure, with a minority of them with specific formats

• ADCs against TAAs of haematological malignancies are more specific than those for solid tumours.

• Niche indications for ADCs included targets for CD71, PSMA, PTK7 or CD74, in breast, lung, stomach or colon cancer.

• Some of these TAAs are essential genes like CD71, PSMA and PTK7

• This study opens the door for further evaluation of ADCs in non‐explored indications.

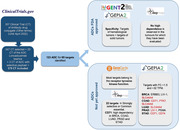

## 1 INTRODUCTION

Identification of novel druggable vulnerabilities in cancer is a major challenge.^1^ Classical approaches aimed to test different chemical entities for antitumoral activity, what has been called hit to lead, and in a second step, lead to hit, to refine its chemical structure.^1^ More recently, the discovery of novel genomic alterations leads to the identification of compounds able to inhibit a specific target protein.^1^, ^2^ For both of these strategies successful examples can be described demonstrating an improvement in clinical outcome.^3^ On the other hand, both approaches are unable to discriminate tumoral cells from non‐transformed ones, if the target is ubiquitously expressed. Moreover, some have shown a narrow therapeutic index with an inadequate safety profile.^1^, ^4^, ^5^

The use of monoclonal antibodies to guide the delivery of active but toxic compounds have demonstrated to be beneficial for patients, with the first approval of antibody‐drug conjugates (ADCs) developed for lymphoma and breast tumours.^6^, ^7^ At this moment, there are 11 ADCs approved^8^ and many others in clinical development.^9^

The successful development of ADCs depends on the correct use of the combination of three structural components, including the design of the antibody against a specific tumour target, the use of a suitable linker, and the selection of an appropriate payload (the cytotoxic component of ADC).^7^, ^10^, ^11^ Regarding the selection of a specific monoclonal antibody, beyond the concrete designing of the antibody itself or the use of specific formats and structures, the identification of a target only expressed within the tumour is key to demonstrating activity with reduced on‐target and off‐tumour toxicity. In this context, the best‐case scenario will be the use of an antibody against a tumour‐associated target (TAT) only expressed in the tumour where the selected payload demonstrated the highest antitumoral activity.^7^

Approved ADCs have demonstrated significant clinical efficacy in increasing time‐to‐event endpoints, like progression‐free survival (PFS) or overall survival (OS) in particular indications, where there is a clear presence of the TAT, as is the case for those targeting HER2 like trastuzumab emtansine and trastuzumab deruxtecan.^12^, ^13^, ^14^ However, a deep evaluation of target expression among all tumour types with the aim of identifying potential clinical options for development has not been performed.

In this article, we evaluate the current development process of ADCs in solid and hematologic malignancies with a special focus on the presence of niche areas for clinical development.

## 2 MATERIAL AND METHODS

### 2.1 Identification and classification of ADC

By using ClinicalTrials.gov (https://www.clinicaltrials.gov/) we performed a search that included private and public‐funded clinical studies. We specified the term “cancer” in the search field “Disease”, and “Antibody‐drug conjugate” in the field “Other terms”. We found 357 clinical trials in this search (22 April 2022). Ten clinical trials were deleted as included compounds to detect and track cancer.

The search was completed including ADCs approved, therefore adding 20 additional clinical trials related to these approved agents. In addition, we added three clinical trials related to ADCs with selective payload. In summary, a total of 370 clinical trials were included in the analysis.

### 2.2 Evaluation of the expression and dependence of ADC targets

Using publicly available datasets (Gene Expression Profiling Interactive Analysis; http://gepia2.cancer‐pku.cn/ using TCGA) (last accessed in July 2022) we analyzed the expression (in transcripts per million, TPM) of these targets in the tumoral and non‐transformed (normal) tissue of all cancer types. We selected those whose TPM was equal to or greater than 32, a value considered medium/high in terms of gene expression.^15^

With the expression data, we calculate the Fold Change between the tumour and normal tissue [(TPM tumour tissue‐TPM normal tissue)/TPM normal tissue]. A Fold Change of 1 means that tumour tissue has twice the expression of normal tissue.

To study the expression by cancer subtypes, also public datasets were interrogated (Gene Expression Database of Normal and Tumor tissues; http://gent2.appex.kr/gent2/) (last accessed in July 2022). Data obtained were normalized by MAS5 and represented by log 2 of the Fold Change to simplify the representation.^16^

The effect on cell viability of target inhibition or suppression was studied using the DepMap portal software tool (https://depmap.org/portal/) (last accessed in July 2022). This tool is used to analyze whether a target is essential for several tumour cell line types (common essential) or for a specific group (strongly selective).

From the score provided by DepMap (Chronos, for CRISPR and DEMETER2, for siRNA), we selected a mean −0.7 score as an arbitrary threshold to choose those genes whose inhibition was closer to being essential. We focused only on those tumours with the highest incidence (Cancer Today‐ WHO; https://gco.iarc.fr/today/home, last accessed June 2022) including breast, colon, lung, gastric and prostate cancer and tumours for which ADCs have been approved. In the case of haematological tumours, non‐Hodgkin's lymphoma is ranked eleventh and leukaemia thirteenth (last accessed June 2023), so they have not been included in this analysis.

## 3 RESULTS

### 3.1 A snapshot of the current clinical development stage of ADCs

**FIGURE 1:**
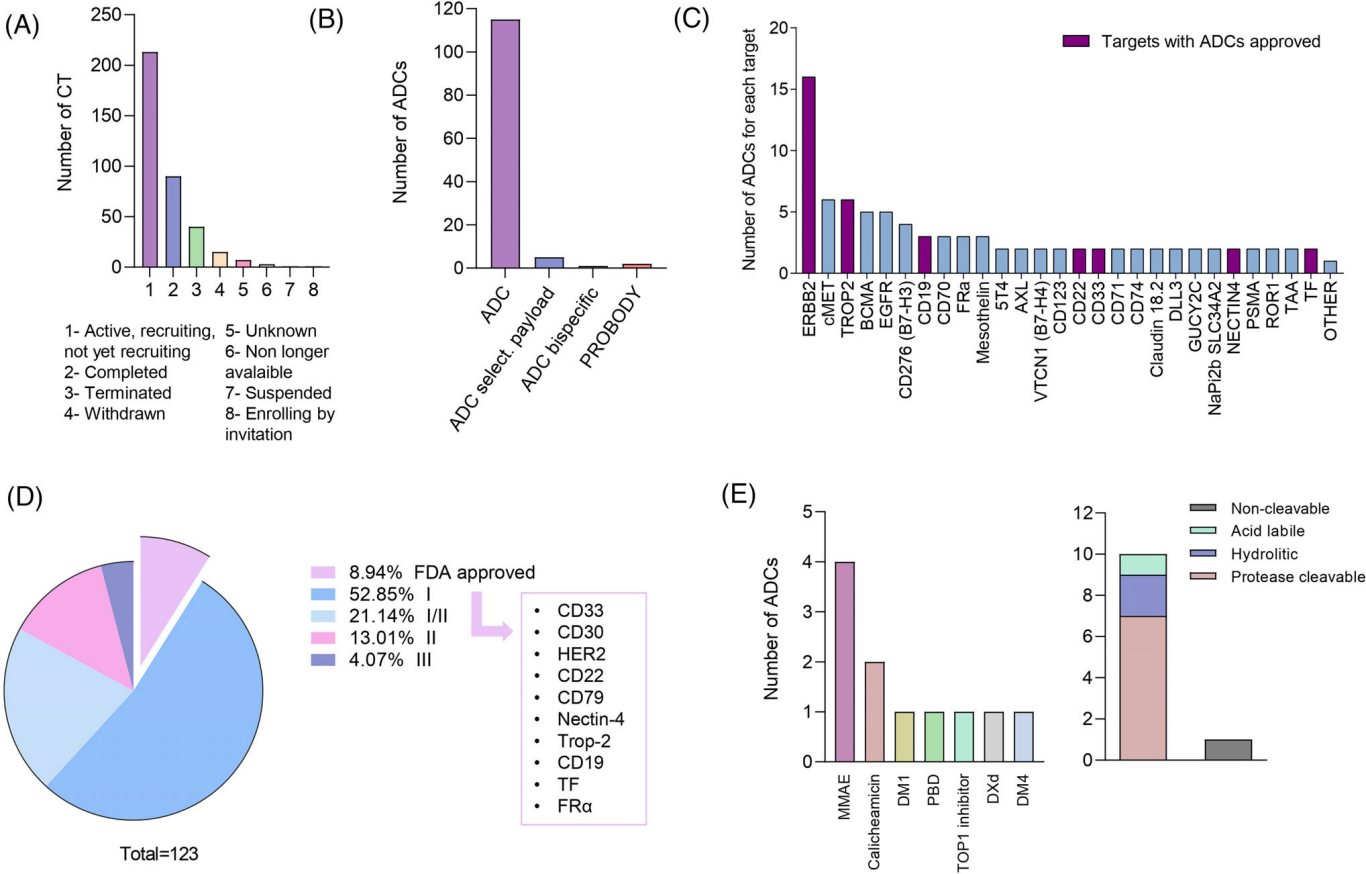
(A) Status of current clinical trials on antibody‐drug conjugates (ADCs). (B) Classification of all ADCs in clinical trials according to their type. (C). Number of ADCs in a clinical trial for each target. Other: ADAM9, AG‐7 antigen, ALCAM, CCR7, CD117 (cKit), CD20, CD205, CD30, CD37, CD38, CD46, CD48, CD56, CD79b, CEACAM5, DPEP3, EFNA4, ENPP3, ERBB3, FGFR2, FGFR3, FLT3, GH, GPNMB, IFG‐1R, Lewis Y antigen, LIV‐1, LRRC15, LYPD3, PTK7, ROR2, SLAMF7, SLC44A4, SLITRK6 and TIM‐1. (D) Classification of ADCs approved and non‐approved (classified according to the phase of the clinical trial). (E) Linkers and payloads of ADCs FDA approved.

Following our research criteria, as described in material and methods, 370 clinical trials (Table S1) were selected and classified into the following subgroups: 213 were reported as active, including those not yet recruiting and currently recruiting; 90 were described as completed; 40 were terminated for several reasons including strategic considerations, lack of efficacy or sponsor prioritization, 15 withdrawn; three were no longer available; one was suspended, and one was available for enrollment only by invitation. Finally, seven of the studies have an “unknown” status (Figure 1A). The clinical efficacy data of approved ADCs^8^ including indications and the corresponding study is displayed in Table S2.

Using those studies as a main source, we identified 123 ADCs. Of these, 115 ADCs had a classical structure (antibody‐linker‐chemotherapy); five had a chemical targeted agent payload (selective inhibitor); two were probody ADCs (designed to remain inactive until proteolytically activated in the tumour microenvironment) and one was a bispecific ADC (antibodies with two binding sites directed at two different antigens) (Figure 1B). The ADCs with a chemical targeted agent payload included ABBV‐155, HDP‐101, BDC‐1001, SBT6050 and NJH395; the probodies included CX‐2029 and CX‐2009 and the bispecific ADC termed M1231 was designed against EGFR/MUC1 (Figure 1B; Table S1). Regarding the tumour antigen target (TAT), most of them were designed against HER2, cMET, TROP2 or BCMA, among others (Figure 1C). Eleven ADCs were FDA‐approved and 112 were at this moment in clinical development (Figure 1D). Within these clinical trials, 18 studies were terminated before completion, for different reasons, being the most frequent the lack of clinical efficacy or strategic considerations (Table S3).

Among those approved, the most frequent targets included CD33, CD30, HER2, CD22, CD79, Nectin‐4, Trop‐2, CD19, Tissue factor and Folate receptor‐α (FRα); and the majority had a MMAE payload (36%), followed by calicheamicin (18%) (Figure 1E). Finally, most of the ADCs had a cleavable linker (91%) compared with those wearing a non‐cleavable one (9%). Among the cleavable ones, those with a protease cleavable linker were the most frequent ones (70%) (Figure 1E).

### 3.2 TATs are highly specific in haematological malignancies

**FIGURE 2:**
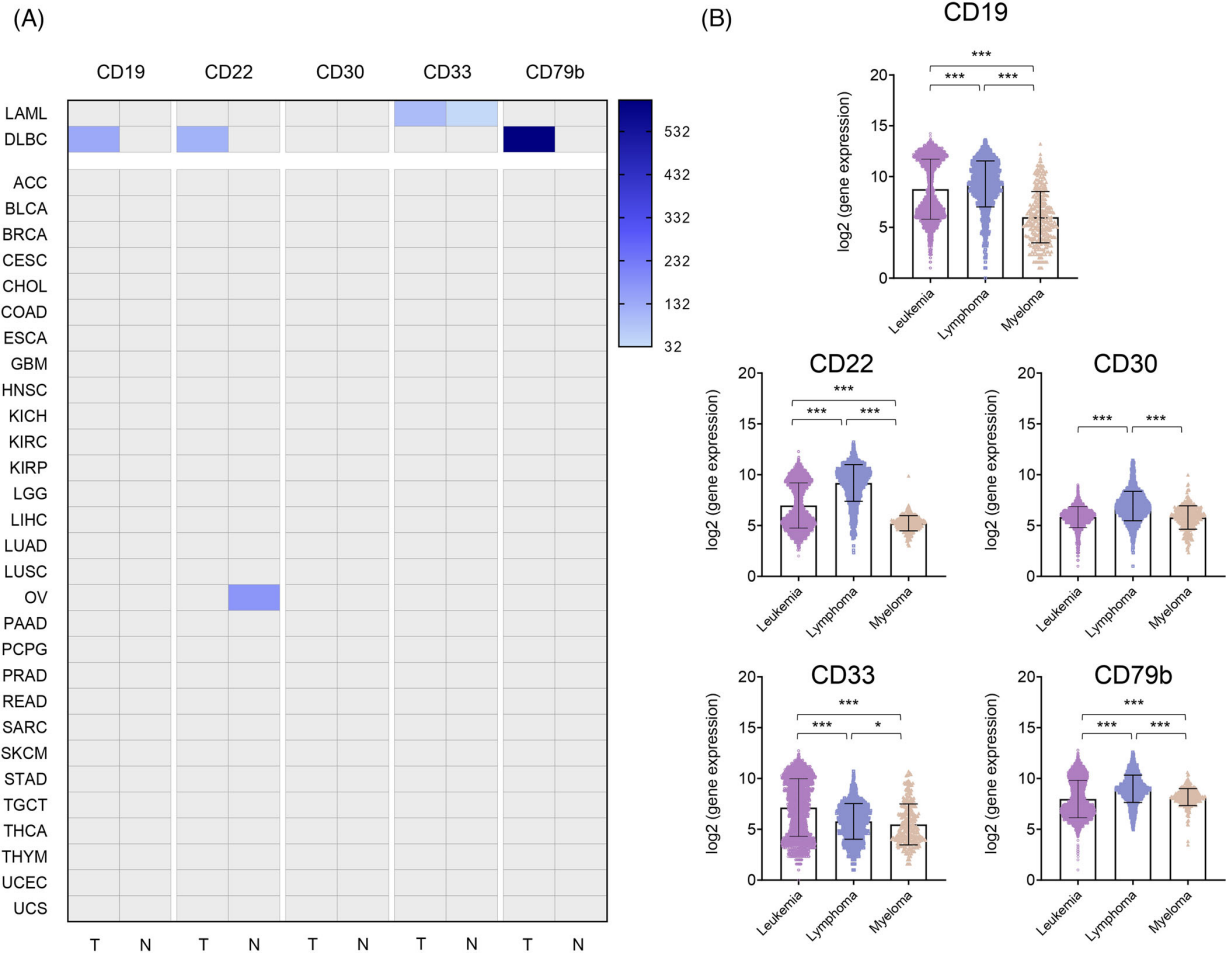
(A) Expression of targets in all tissues of antibody‐drug conjugates (ADCs) FDA‐approved used for haematological tumours (GEPIA2). (B) Target expression according to haematological tumour type (GENT2).

ADCs targeting BCMA are approved in Multiple Myeloma, CD19 in Diffuse large B cell lymphoma (DLBCL) and CD22 in Acute B Lymphoblastic Leukemia (B‐ALL) (Table 1). Using genomic data (Table S4) to evaluate the expression of TATs, as described in the material and methods section, we observed that, TATs expressed in haematological malignancies were highly specific and not expressed in normal tissue or other different tumour types (Figure 2A). Only one exception was identified involving the presence at a genomic level of *CD22* in normal ovarian tissue (Figure 2A).

**TABLE 1:** Characteristics of antibody‐drug conjugates (ADCs) currently approved by the FDA for haematological tumours.

ADC	Target	Linker	Payload	Condition
Loncastuximab Tesirine	CD19	A cleavable (valine‐alanine dipeptide as cathepsin B cleavage site) maleimide type linker containing a hematológicos spacer PEG	PBD	DLBCL
Inotuzumab ozogamicin	CD22	Acid‐labile 4‐(4′‐acetylphenoxy) butanoic acid (acetyl butyrate) linker	Calicheamicin	B‐ALL
Brentuximab vedotin	CD30	Protease‐cleavable linker	MMAE	ALCL, cHL
Gemtuzumab Ozogamicin	CD33	Hydrolytic cleavage bifunctional linker (4‐(4‐acetylphenoxy)butanoic acid)	Calicheamicin	AML
Polatuzumab Vedotin	CD79b	Protease‐cleavable peptide linker (valine–citrulline; Maleimidocaproylvaline‐citrulline‐p‐aminobenzoyloxycarbonyl or MC‐VC‐PABC)	MMAE	DLBCL

We observed that some of the described targets and approved ADCs in the mentioned indications, including *CD19, CD22, CD30, CD33* and *CD79b*, were also present in other haematological tumours (Figure 2A). In line with this observation, ADCs against these targets are under clinical evaluation in other haematological tumour types (Table S5).

Table 1 shows a summary of the ADCs approved for the treatment of haematological tumours, including their target, linker and payload, as well as the main approved indication. As can be seen in Figure 2B, *CD79B* is also highly expressed in myeloma, which suggests that acting on this receptor would be a reasonable therapeutic opportunity that is not currently in clinical evaluation (Table 1 and Table S5).

### 3.3 Mapping TATs in solid tumours to identify niche areas for clinical development

**FIGURE 3:**
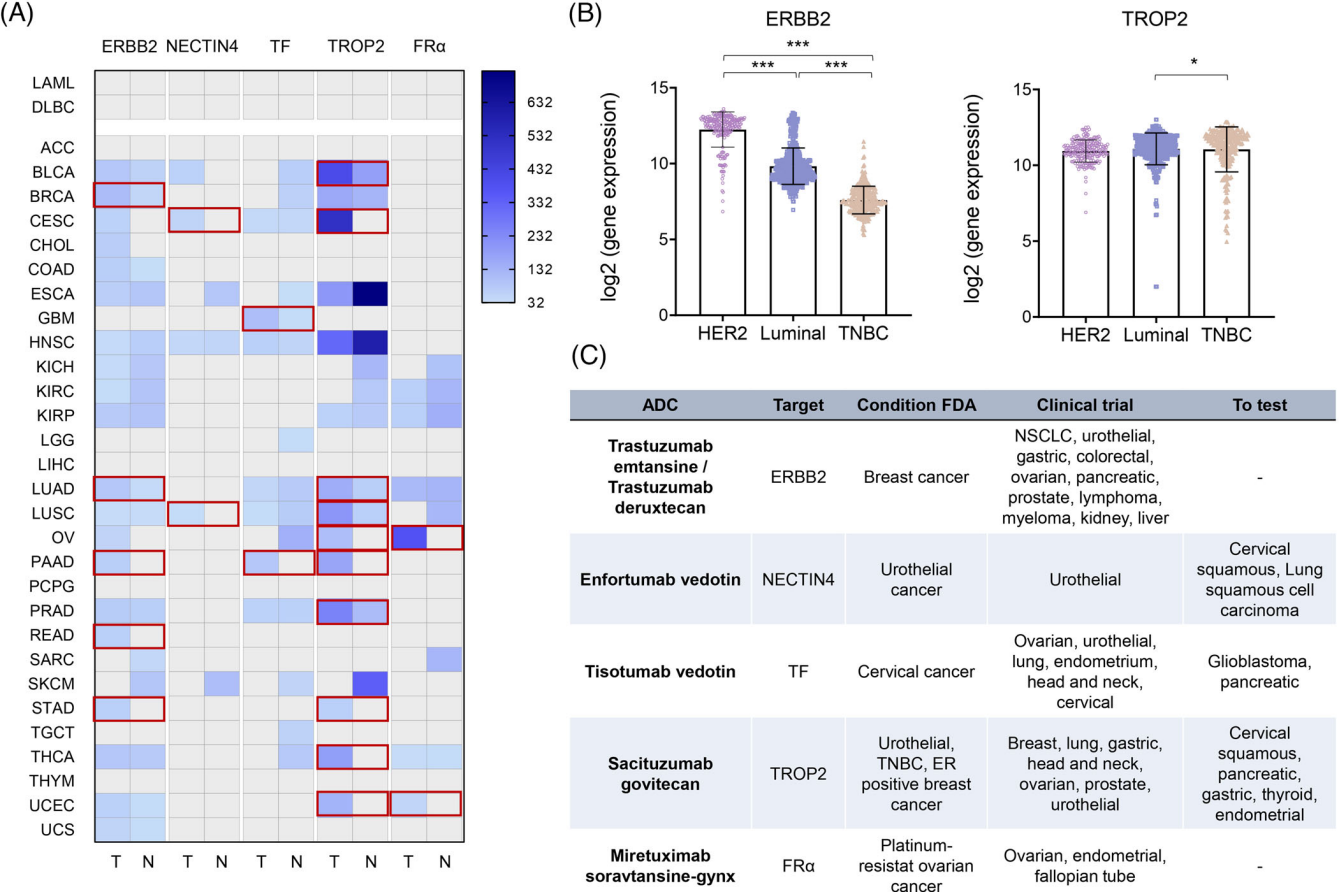
(A) Expression of targets in all tissues of antibody‐drug conjugates (ADCs) FDA‐approved used for solid tumours (GEPIA2). (B) Expression of ERBB2 and TROP2 according to breast cancer subtype (GENT2). (D) Table with characteristics of approved ADCs for solid tumours.

Next, we used genomic data to evaluate the presence of the TAT for approved ADC, in different indications including solid tumours, diffuse large B cell lymphoma and acute myeloid leukaemia. Highlighted in red, in Figure 3A, we can observe those indications for which the target is overexpressed in tumour tissue with a statistically significant difference. *TROP2* is overexpressed in several tumour types including bladder, cervix, lung, ovarian, pancreas, prostate, stomach and thyroid. Of note, high expression is also observed in normal tissues including oesophagus tissue and skin (Figure 3A). Side effects associated with the drug Sacituzumab govitecan have been reported including itching, rash, alopecia, mucosal inflammation and coughing among others, that could be related to the presence of the target in those mentioned tissues.^17^ In the case of TATs for breast cancer, we observed that *ERBB2* was exclusively expressed in the so‐called HER2‐positive breast cancer, due to the presence of an amplified gene (Figure 3B). However, for *TROP2*, a significant presence was also observed in luminal and HER2‐positive tumours (Figure 3B). In a similar way, among tumours for which TATs were highly expressed, we found some indications where these ADCs were not under clinical evaluation. Figure 3C summarizes some of the data including options for clinical development such as the use of Enfortumab vedotin (Nectin‐4) in cervical and lung cancer; Tisotumab vedotin (TF) in glioblastoma and pancreatic cancer; or Sacituzumab govitecan (TROP2) in cervical, pancreatic, gastric, thyroid, and endometrial cancer. In line with this, Sacituzumab govitecan has recently received US FDA approval in ER‐positive breast cancer^18^ (Figure 3B).

### 3.4 Some TATs are common essential genes

**FIGURE 4:**
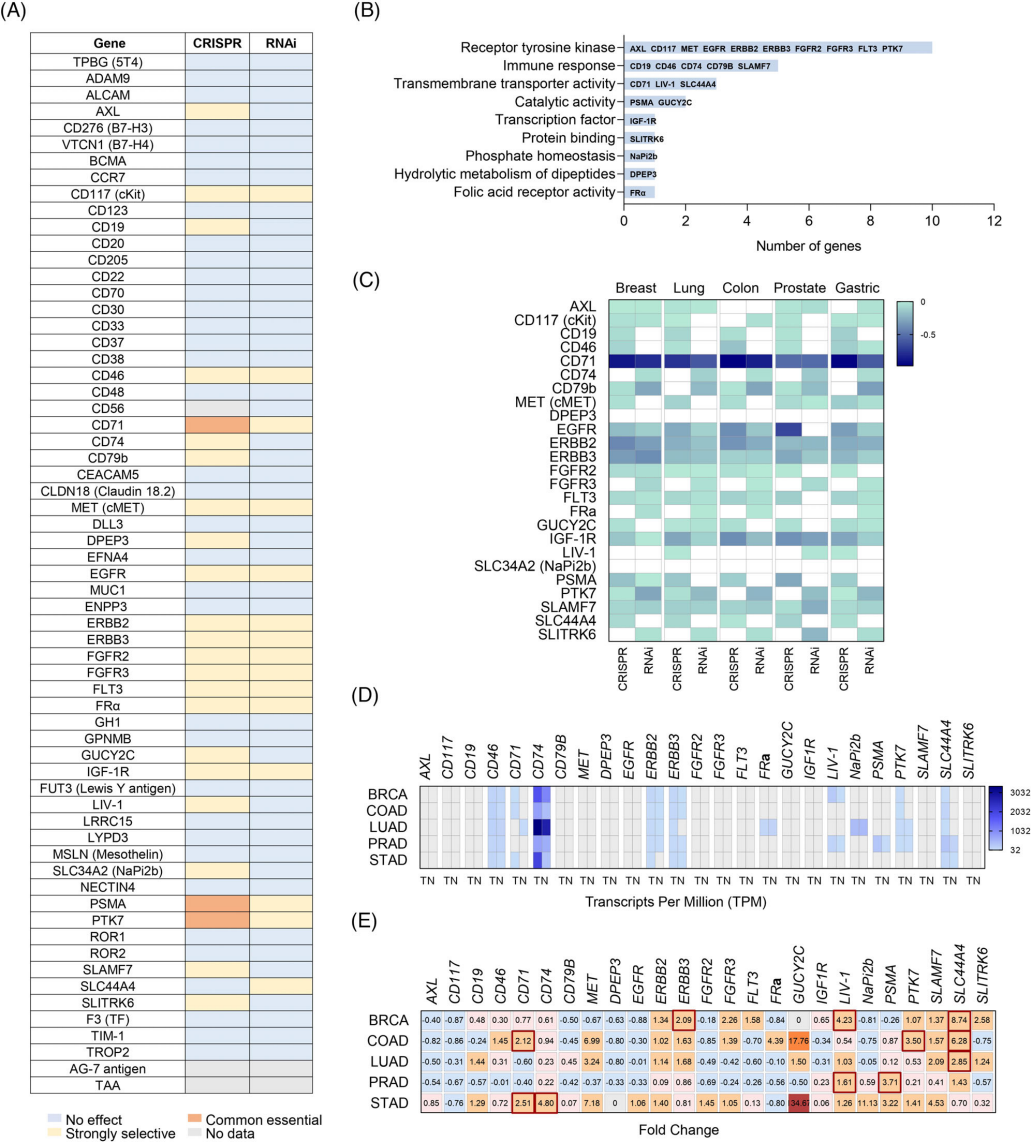
(A) Effect of silencing by both CRISPR and RNAi on different cell lines from several tumour types. Data from DepMap portal. The scores appraise the effect size of knocking down or knocking out human genes. A negative score indicates that the cell lines grow slower after knocking down or knocking out a gene, while a positive score indicates that the cell lines grow faster. Common essential: A gene which, in a large, pan‐cancer screen,^20^ ranks in the top most depleting genes in at least 90% of cell lines (the score of these genes are used as the dependent distribution for inferring dependency probability). Strongly selective: a gene whose dependency is at least 100 times more likely to have been sampled from a skewed distribution than a normal distribution. (B) Functions of targets dependent on cell lines. (C) Chronos and DEMETER2 dependency score of each gene in the five tumours with the highest incidence including breast, lung, colorectal, prostate and gastric cancer. A score of zero indicates that a gene is not essential, and a score of −1 is comparable to the median of all pan‐essential genes. (D) RNA expression in TPM according to GE PIA2 of targets in five tumours with the highest incidence. A value of 32 TPM is considered a medium‐high expression. (E) Fold Change (FC) data between normal vs tumour tissue for the above genes in the five tumours with the highest incidence. The FC has been calculated in such a way that a value of 0 means equal expression, and a value of 1 means double expression in tumour tissue. FC greater than 1.5, and with expression more than 32 TPM are marked in red.

We next evaluated if some of the evaluated TATs played an oncogenic role, being targets themselves. In this context, the anti‐tumour effect of the ADC will not be only produced by the action of the payload, but through the inactivation of the oncogenic action of the membrane receptor. To do this, we used a bioinformatics approach, as described in the material and methods section. We identified three targets *CD71*, *PSMA* and *PTK7* that were commonly essential. This means that their down‐regulation would affect tumour cell proliferation (Figure 4A). In a less significant manner, we identified as “strongly selective” the following targets: *AXL, CD117, CD46, CD74, CD79b, MET, DPEP3, EGFR, ERBB2, ERBB3, FGFR2, FGFR3, FLT3, FRα, GUCY2C, IGF‐1R, LIV‐1, NaPi2b*, *SLAMF7, SLC44A4* and *SLITRK6* (Figure 4A). We also analyzed the score for deletion (CRISPR) of each target in the tumours for which they were evaluated (Figure S1A). We did not observe a high dependence in these tumours, except for *CD71*, in haematological tumours (Figure S1A). Commonly essential and strongly selective genes belonged principally to the receptor tyrosine kinase and immune function, as can be seen in Figure 4B. We observed that *CD71* is one of the most essential targets in breast, colon and gastric tumours (Figure 4C). Other common essential targets include *EGFR* in prostate cancer (Figure 4C).

### 3.5 Expression of common essential genes in most prevalent tumours

The expression of the identified common essential TATs was evaluated using genomic data in the five more prevalent tumours including breast (BRCA), colorectal (COAD), lung (LUAD), prostate (PRAD) and gastric (STAD). A significant upregulation compared with normal tissue was observed for *CD74* for breast, colorectal and gastric tumours and *CD71* for breast, lung or stomach as can be seen in Figure 4D. In addition, *PTK7* and *SLC44A4* showed high expression in breast and colon tumours (Figure 4D). To complete the analysis, we studied those genes that had a Fold Change (FC) greater than 1.5 with 32 TPM (Figure 4E). Genes identified in breast cancer included *ERBB3, LIV‐1* and *SLC44A4*; in colorectal cancer *CD71*, *PTK7* and *SLC44A4*; in lung cancer *SLC44A4*; in prostate cancer *LIV‐1* and *PSMA*; and finally in stomach cancer *CD71* and *CD74* (Figure 4E).

As in our previous analysis, we matched if some of these indications were not clinically exploited. Thus, ADCs directed against SLC44A4 could potentially be evaluated in breast cancer; CD71, PTK7 and SLC44A4 in colorectal cancer; SLC44A4 in lung cancer; LIV‐1 in prostate cancer, and finally CD71 and CD74 in gastric cancer. This data suggests potential opportunities for development in the described indications (Figure S1B).

## 4 DISCUSSION

In the present study, we evaluate the current clinical development of ADCs in solid tumours and some haematological malignancies. By extracting the data of the ongoing clinical studies, we aimed to have a snapshot of the current development to improve the clinical development of these agents.

We first observed that most of the ADCs in development were based on a classical antibody structure and only a minority included nanobodies or bispecific formats. This clearly shows the limitations that these types of formats can display at the production and manufacturing level, and explain why clinical implementation is gradual. When analyzing TATs, we observed that most of them included well‐known targets such as HER2, cMET, TROP2 or BCMA. This data highlights the clear bottleneck that represents for this family of compounds the identification of novel targets, and the clear opportunity that represents the discovery of new surfaceome proteins. Most used payloads and linkers included MMAE and cleavable linkers, respectively. Of note, old targets like CD20 have hardly been implemented as ADCs. A potential reason could be the lack of differentiation compared with new competitor ADCs in the same indication.

When evaluating TATs, we observed a high specificity of the target in haematological malignancies compared with normal tissue, and some haematological indications shared the expression of the target. In this context, some opportunities for development could be exploited like targeting CD79b in multiple myeloma. In contrast, TATs in solid tumours were widely present in several indications and in normal tissue demonstrating a lack of specificity. For instance, TROP2 is overexpressed in several tumour types including bladder, cervix, lung, ovarian, pancreas, prostate, stomach and thyroid, but also in normal tissues including oesophagus tissue and skin, which suggests a potential narrow therapeutic index. When evaluating opportunities for development in indications with expression of the TAT, we identified several scenarios not exploited like Enfortumab vedotin, anti‐Nectin4, in cervical and lung cancer, Tisotumab vedotin, anti‐TF, in glioblastoma and pancreatic cancer, or Sacituzumab govitecan, anti‐TROP2, in cervical, pancreatic, gastric, thyroid and endometrial cancer. To the best of our knowledge, none of these opportunities is under clinical evaluation.

Of note, we identified that some of the TATs selected were also oncogenic proteins that if downregulated could have an antitumor role. To do so, we used a standard bioinformatics approach as described elsewhere.^19^, ^20^ Examples included CD71, PSMA and PTK7. We also identified that ADCs against CD71, PTK7, CD74, SLC44A4 and LIV‐1 were not under evaluation in breast, colorectal, prostate and gastric cancer, which suggests potential opportunities for evaluation. A further step will be the identification of indications to evaluate combinations including those with novel immunotherapies where relevant preclinical data has been reported.^21^, ^22^, ^23^

Among important aspects to take into consideration and that could influence ADC activity, we can refer to some of the following: the oncogenic role of the receptor, antibody binding, internalization of the receptor, recycling, linker cleavage, lysosomal processing, payload diffusion through the lysosomal and cellular membranes,^11^ or the particular antitumor activity to the payload in specific cancers. In this context, spatial tumour heterogeneity and drug penetration are key concepts, particularly for this kind of compound, that depend on the presence of the target, in addition to their very high molecular weight.^24^, ^25^ ADCs with a cleavage linker and with adequate physico‐chemical properties of the payload can have a much better bystander effect and potentially overcome some of these problems.

The main limitation of this study is related to the evaluation of the targets at a transcriptomic level without including protein data. Of note, the lack of datasets of protein expression with reliable information for the evaluated genes made this objective unrealistic.

## 5 CONCLUSIONS

In the current article, we describe novel opportunities for drug development of approved and under clinical evaluation ADCs. For instance, agents against some overexpressed targets such as CD71, PTK7, CD74 and SLC44A4 could be evaluated in some conditions such as breast, colon and gastric cancer. In summary, we provide a snapshot of the current landscape of ADCs in cancer providing information about potential new options to exploit clinically.

## CONFLICT OF INTEREST STATEMENT

The authors declare no conflict of interest.

## Supporting information


**Figure S1** (A) DepMap scoring of previously identified targets for various cell lines. Here, we analyze dependence on the tumours for which they have been tested in clinical trials. (B) Summary table of genes that are highly expressed in tumour tissue compared to normal tissue. We represent the payload, tumours for which it is evaluated in clinical trials and tumours for which it should be evaluated.

**Table S1** Clinical trials of all currently evaluated ADCs.

**Table S2** Efficacy of approved ADCs.

**Table S3** Summary table of clinical trials that were terminated prematurely.

**Table S4** Number of samples for each tumour and normal tissue that have been genomically analyzed (TCGA and GTEx)

**Table S5** ADCs targeting haematological tumours.

## Data Availability

All data generated or analyzed during this study are included in this published article (and its supplementary information files).
