# Supplementary figures and images for: Uncovering therapeutic opportunities in the clinical development of antibody‐drug conjugates
Source: Clin Transl Med. 2023 Sep 22;13(9):e1329. doi: 10.1002/ctm2.1329 (PMC10517221; doi:10.1002/ctm2.1329)

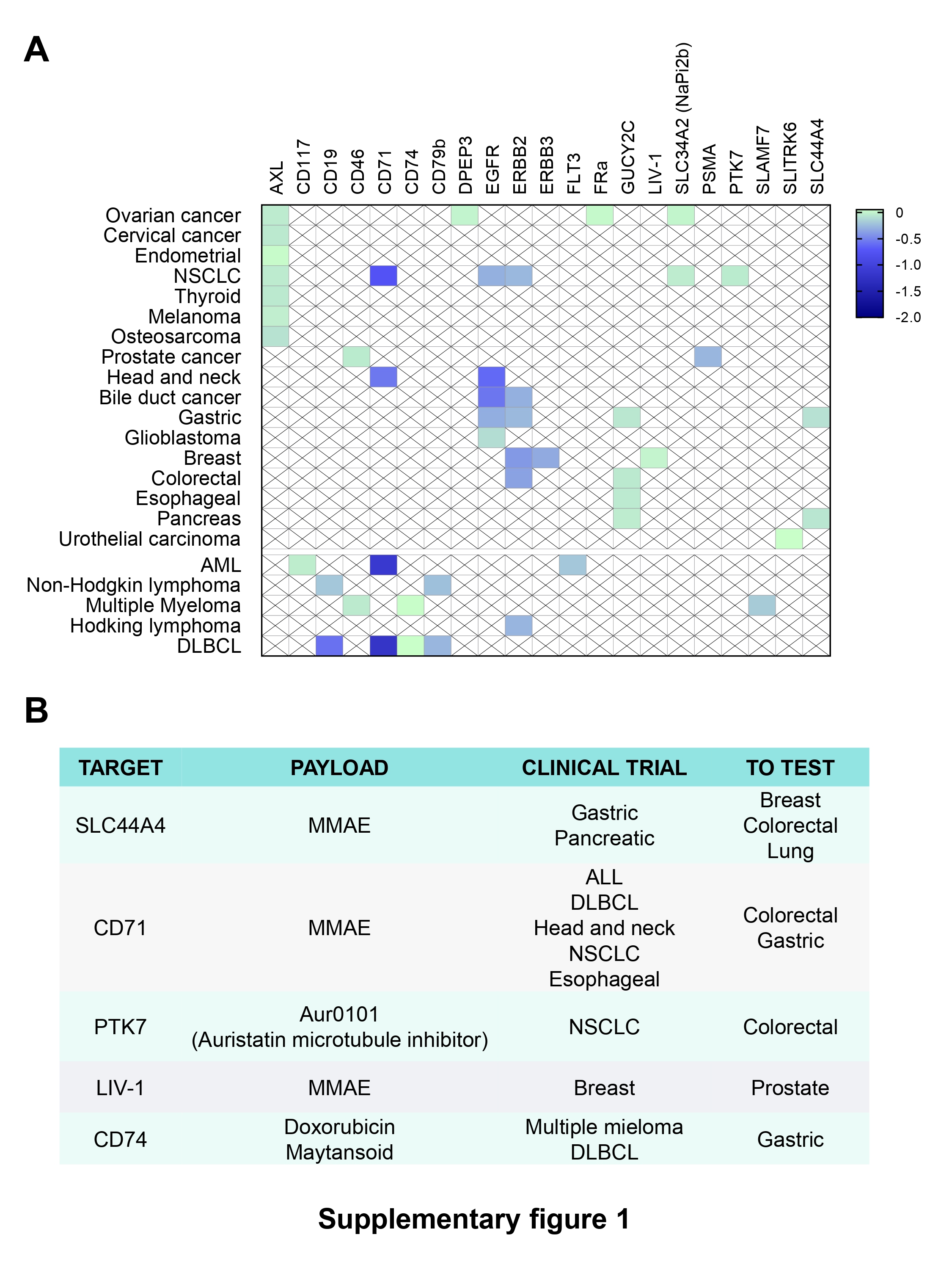

Supplement: Supplementary file 1 — Figure S1 (A) DepMap scoring of previously identified targets for various cell lines. Here, we analyze dependence on the tumours for which they have been tested in clinical trials. (B) Summary table of genes that are highly expressed in tumour tissue compared to normal tissue. We represent the payload, tumours for which it is evaluated in clinical trials and tumours for which it should be evaluated. [file CTM2-13-e1329-s003.jpg]

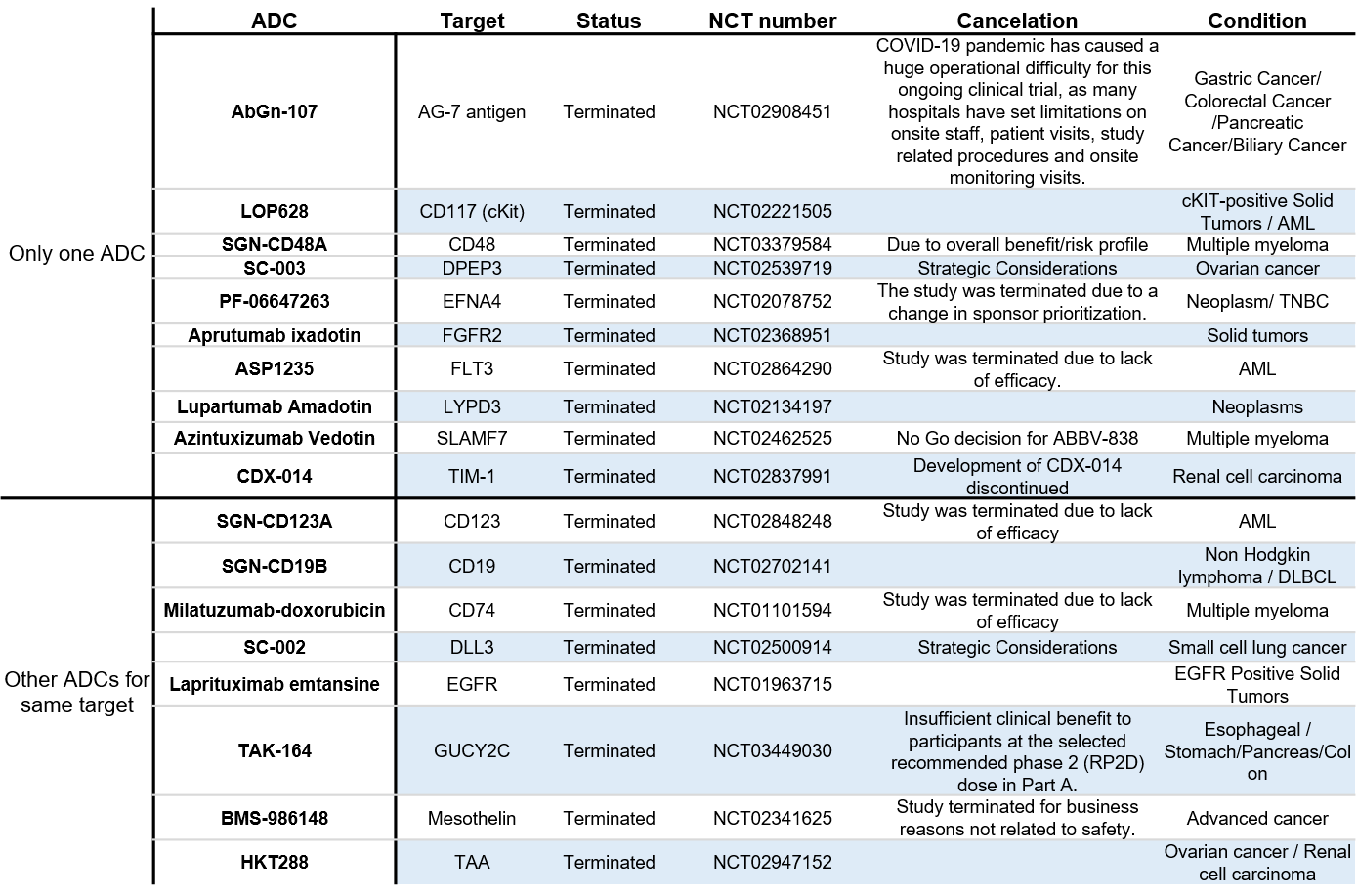


**Supplementary table 3**

Supplement: Supplementary file 4 — Table S3 Summary table of clinical trials that were terminated prematurely. [file CTM2-13-e1329-s005.docx]

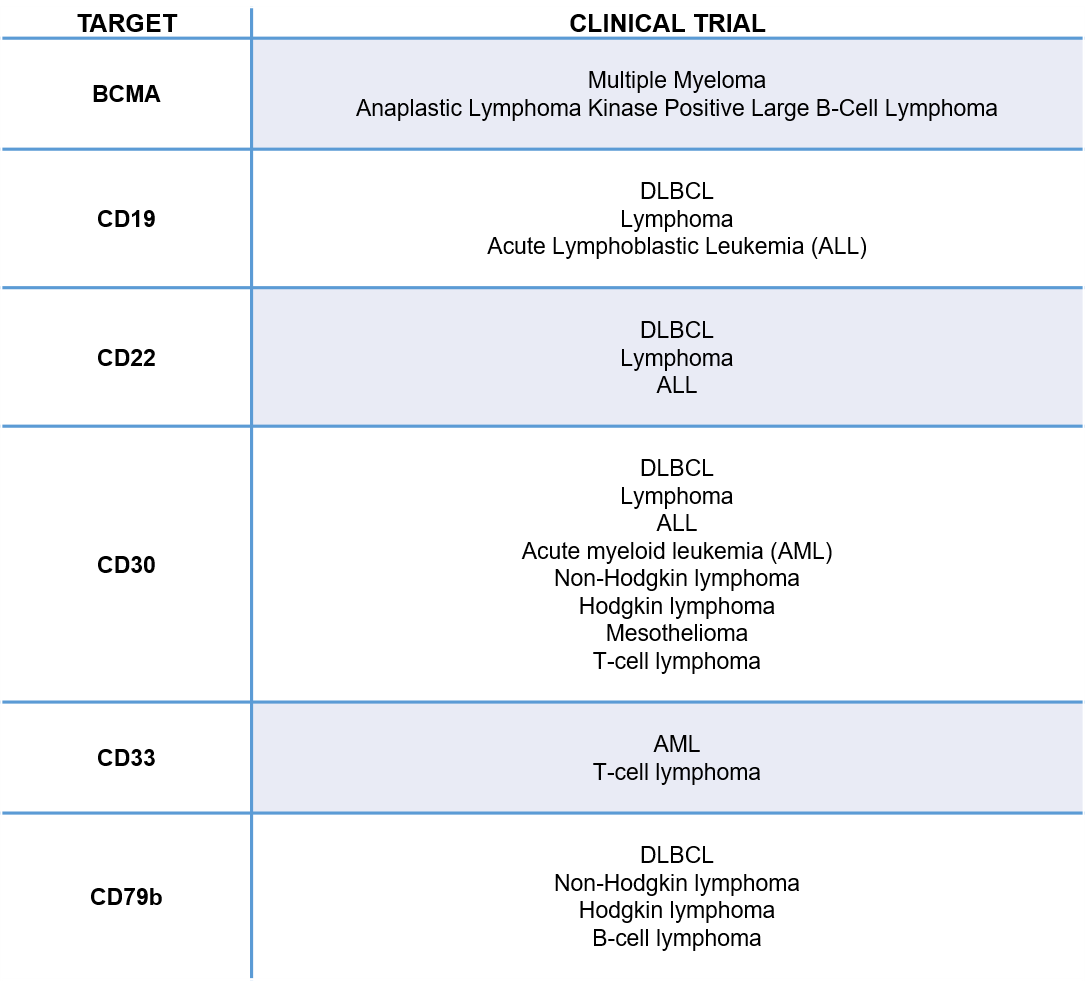


**Supplementary table 5**

Supplement: Supplementary file 6 — Table S5 ADCs targeting haematological tumours. [file CTM2-13-e1329-s004.docx]
