# Supplementary material for: Uncovering therapeutic opportunities in the clinical development of antibody‐drug conjugates
Source: Clin Transl Med. 2023 Sep 22;13(9):e1329. doi: 10.1002/ctm2.1329 (PMC10517221; doi:10.1002/ctm2.1329)
Supplement: Supplementary file 2 — Table S1 Clinical trials of all currently evaluated ADCs. [file CTM2-13-e1329-s006.docx]

| ADC | Target | Payload | Status | Phases | NCT Number |
| --- | --- | --- | --- | --- | --- |
| ASN-004 | 5T4 | Auristatin F hydroxypropylamide | Not yet recruiting | Phase 1 | NCT04410224 |
| SYD1875 | 5T4 | Duocarmycin analogues | Active, not recruiting | Phase 1 | NCT04202705 |
| IMGC936 | ADAM9 | DM21 | Recruiting | Phase 1\|Phase 2 | NCT04622774 |
| AbGn-107 | AG-7 antigen | DM4 | Terminated | Phase 1 | NCT02908451 |
| CX-2009 | ALCAM | DM4 | Completed | Phase 1\|Phase 2 | NCT03149549 |
| Enapotamab vedotin | AXL | MMAE | Completed | Phase 1\|Phase 2 | NCT02988817 |
| Mecbotamab Vedotin | AXL | MMAE | Recruiting | Phase 2 | NCT04681131 |
|  |  |  | Recruiting | Phase 1\|Phase 2 | NCT03425279 |
| DS-7300a | B7-H3 | DXd | Not yet recruiting | Phase 2 | NCT05280470 |
| HS-20093 | B7-H3 | - | Recruiting | Phase 1 | NCT05276609 |
| MGC018 | B7-H3 | Synthetic duocarmycin analogs (pro-drug seco-duocarmycin-p-hydroxybenzamide-azaindole (seco-DUBA) | Recruiting | Phase 1\|Phase 2 | NCT03729596 |
|  |  |  | Recruiting | Phase 1 | NCT05293496 |
| AZD8205 | B7-H4 | TOP1i | Recruiting | Phase 1\|Phase 2 | NCT05123482 |
| HS-20089 | B7-H4 | - | Recruiting | Phase 1 | NCT05263479 |
| Belantamab mafodotin | BCMA | MMAF | Recruiting | Phase 1\|Phase 2 | NCT04126200 |
|  |  |  | Active, not recruiting | Phase 2 | NCT03544281 |
|  |  |  | Active, not recruiting | Phase 1 | NCT03828292 |
|  |  |  | Active, not recruiting | Phase 2 | NCT03525678 |
|  |  |  | Completed | Phase 1 | NCT04177823 |
|  |  |  | Recruiting | Phase 1\|Phase 2 | NCT04822337 |
|  |  |  | Recruiting | Phase 2 | NCT04680468 |
|  |  |  | Recruiting | Phase 1 | NCT04398745 |
|  |  |  | Recruiting | Phase 2 | NCT05064358 |
|  |  |  | Recruiting | Phase 2 | NCT04676360 |
| CC-99712 | BCMA | Maytansinoid | Recruiting | Phase 1 | NCT04036461 |
| GSK2857916 | BCMA | MMAF | Completed | Phase 1 | NCT02064387 |
| MEDI2228 | BCMA | Pyrrolobenzodiazepine (PBD) | Active, not recruiting | Phase 1 | NCT03489525 |
| HDP-101 | BCMA | RNA pol II inhibitor | Recruiting | Phase 1\| Phase 2 | NCT04879043 |
| JBH492 | CCR7 | - | Recruiting | Phase 1 | NCT04240704 |
| LOP628 | CD117 (cKit) | Maytansine | Terminated | Phase 1 | NCT02221505 |
| IMGN632 | CD123 | Sulfonated DGN462 | Recruiting | Phase 1\|Phase 2 | NCT03386513 |
|  |  |  | Not yet recruiting | Phase 1\|Phase 2 | NCT05320380 |
| SGN-CD123A | CD123 | PBD | Terminated | Phase 1 | NCT02848248 |
| Denintuzumab mafodotin | CD19 | MMAF | Terminated | Phase 2 | NCT02592876 |
|  |  |  | Terminated | Phase 2 | NCT02855359 |
|  |  |  | Completed | Phase 1 | NCT01786135 |
|  |  |  | Completed | Phase 1 | NCT01786096 |
| Loncastuximab Tesirine | CD19 | PBD | Completed | Phase 1 | NCT02669017 |
|  |  |  | Terminated | Phase 1 | NCT02669264 |
|  |  |  | Terminated | Phase 1 | NCT03685344 |
|  |  |  | Active, not recruiting | Phase 2 | NCT03589469 |
|  |  |  | Recruiting | Phase 1\|Phase 2 | NCT03684694 |
|  |  |  | Not yet recruiting | Phase 2 | NCT05249959 |
|  |  |  | Not yet recruiting | Phase 1 | NCT05053659 |
|  |  |  | Active, not recruiting | Phase 2 | NCT04699461 |
|  |  |  | Recruiting | Phase 3 | NCT04384484 |
|  |  |  | Recruiting | Phase 2 | NCT04998669 |
|  |  |  | Not yet recruiting | Phase 2 | NCT05144009 |
|  |  |  | Not yet recruiting | Phase 2 | NCT05222438 |
|  |  |  | Not yet recruiting | Phase 2 | NCT05296070 |
|  |  |  | Not yet recruiting | Phase 1 | NCT04970901 |
|  |  |  | Withdrawn | Phase 1 | NCT04974996 |
|  |  |  | Recruiting | Phase 2 | NCT05190705 |
|  |  |  | Recruiting |  | NCT05160064 |
|  |  |  | No longer available |  | NCT04705454 |
|  |  |  | Not yet recruiting | Phase 1 | NCT05228249 |
|  |  |  | Not yet recruiting | Phase 1 | NCT05270057 |
| SGN-CD19B | CD19 | PBD | Terminated | Phase 1 | NCT02702141 |
| MRG001 | CD20 | MMAE | Recruiting | Phase 1 | NCT05155839 |
| OBT076 | CD205 | DM4 | Recruiting | Phase 1 | NCT04064359 |
|  |  |  | Terminated | Phase 1 | NCT03403725 |
| Inotuzumab ozogamicin | CD22 | Calicheamicin | Recruiting | Phase 2 | NCT03913559 |
|  |  |  | Completed |  | NCT04456959 |
|  |  |  | Active, not recruiting | Phase 4 | NCT03677596 |
|  |  |  | Withdrawn | Phase 3 | NCT03628053 |
| TRPH-222 | CD22 | Maytansine | Recruiting | Phase 1 | NCT03682796 |
| ABBV-155 | CD276 | BCL-xL inhibitor | Recruiting | Phase 1 | NCT03595059 |
| Brentuximab vedotin | CD30 | MMAE | Withdrawn | Phase 1\|Phase 2 | NCT01620229 |
|  |  |  | Withdrawn | Phase 1 | NCT02780011 |
|  |  |  | Completed | Phase 2 | NCT01461538 |
|  |  |  | Completed | Phase 1 | NCT01830777 |
|  |  |  | Completed | Phase 1 | NCT01309789 |
|  |  |  | Completed | Phase 1 | NCT00430846 |
|  |  |  | Terminated | Phase 1 | NCT00649584 |
|  |  |  | Completed | Phase 1 | NCT01026415 |
|  |  |  | Unknown status | Phase 2 | NCT01476410 |
|  |  |  | Completed | Phase 2 | NCT01393717 |
|  |  |  | Completed | Phase 2 | NCT00947856 |
|  |  |  | Completed | Phase 1 | NCT01026233 |
|  |  |  | Recruiting | Phase 2 | NCT01703949 |
|  |  |  | Completed | Phase 4 | NCT01990534 |
|  |  |  | Recruiting | Phase 2 | NCT03007030 |
|  |  |  | Active, not recruiting | Phase 1\|Phase 2 | NCT02227199 |
|  |  |  | Completed | Phase 2 | NCT02505269 |
|  |  |  | Not yet recruiting | Phase 2 | NCT04795869 |
|  |  |  | Completed | Phase 2 | NCT01534078 |
|  |  |  | Completed | Phase 2 | NCT02280785 |
|  |  |  | Active, not recruiting | Phase 2 | NCT01508312 |
|  |  |  | Recruiting | Phase 2 | NCT03540849 |
|  |  |  | Completed | Phase 2 | NCT02567851 |
|  |  |  | Completed | Phase 2 | NCT02388490 |
|  |  |  | Withdrawn | Phase 2 | NCT02623920 |
|  |  |  | Completed | Phase 2 | NCT01396070 |
|  |  |  | Recruiting | Phase 2 | NCT02588651 |
|  |  |  | Withdrawn | Not Applicable | NCT01671813 |
|  |  |  | Recruiting | Phase 2 | NCT04378647 |
|  |  |  | Completed | Phase 2 | NCT01807598 |
|  |  |  | Completed | Phase 1\|Phase 2 | NCT01492088 |
|  |  |  | Completed | Phase 2 | NCT01421667 |
|  |  |  | Active, not recruiting | Phase 2 | NCT02298257 |
|  |  |  | Completed | Phase 1 | NCT02822586 |
|  |  |  | Terminated | Phase 1\|Phase 2 | NCT01805037 |
|  |  |  | Active, not recruiting | Phase 3 | NCT01712490 |
|  |  |  | Completed | Phase 1 | NCT01950364 |
|  |  |  | Terminated | Not Applicable | NCT01841021 |
|  |  |  | Completed | Phase 1\|Phase 2 | NCT02572167 |
|  |  |  | Completed | Phase 1 | NCT01060904 |
|  |  |  | Completed | Phase 3 | NCT01100502 |
|  |  |  | Completed | Phase 1\|Phase 2 | NCT01874054 |
|  |  |  | Completed | Phase 2 | NCT00848926 |
|  |  |  | No longer available |  | NCT01196208 |
|  |  |  | Recruiting | Phase 2 | NCT04561206 |
|  |  |  | Recruiting | Phase 2 | NCT03409432 |
|  |  |  | Active, not recruiting | Phase 2 | NCT03057795 |
|  |  |  | Active, not recruiting | Phase 2 | NCT02744612 |
|  |  |  | Active, not recruiting | Phase 2 | NCT02758717 |
|  |  |  | Completed | Phase 1\|Phase 2 | NCT01780662 |
|  |  |  | Terminated | Phase 1 | NCT02254239 |
|  |  |  | Completed | Phase 1 | NCT01902160 |
|  |  |  | Recruiting | Phase 2 | NCT01716806 |
|  |  |  | Terminated | Phase 2 | NCT01925612 |
|  |  |  | Completed | Phase 3 | NCT01777152 |
|  |  |  | Completed | Phase 2 | NCT00866047 |
|  |  |  | Active, not recruiting | Phase 4 | NCT01909934 |
|  |  |  | Withdrawn | Phase 1\|Phase 2 | NCT02729961 |
|  |  |  | Recruiting | Phase 2 | NCT04587687 |
|  |  |  | Withdrawn | Phase 1 | NCT03373305 |
|  |  |  | Completed | Phase 2 | NCT02939014 |
|  |  |  | Recruiting | Phase 1\|Phase 2 | NCT01896999 |
|  |  |  | Not yet recruiting | Phase 2 | NCT05039073 |
|  |  |  | Active, not recruiting | Phase 1 | NCT03013933 |
|  |  |  | Active, not recruiting | Phase 1\|Phase 2 | NCT01771107 |
|  |  |  | Recruiting | Phase 2 | NCT03233347 |
|  |  |  | Active, not recruiting | Phase 3 | NCT02166463 |
|  |  |  | Recruiting | Phase 2 | NCT04745949 |
|  |  |  | Recruiting | Phase 3 | NCT03907488 |
|  |  |  | Active, not recruiting | Phase 2 | NCT03113500 |
|  |  |  | Recruiting | Phase 2 | NCT03712202 |
|  |  |  | Active, not recruiting | Phase 2 | NCT01979536 |
|  |  |  | Not yet recruiting | Phase 2 | NCT05313243 |
| Gemtuzumab Ozogamicin | CD33 | Calicheamicin | Active, not recruiting | Phase 1\|Phase 2 | NCT03531918 |
|  |  |  | Recruiting | Phase 2 | NCT03374332 |
|  |  |  | No longer available | - | NCT02312037 |
| Vadastuximab talirine | CD33 | PBD | Terminated | Phase 1\|Phase 2 | NCT02614560 |
|  |  |  | Terminated | Phase 3 | NCT02785900 |
|  |  |  | Completed | Phase 1 | NCT02326584 |
|  |  |  | Terminated | Phase 1\|Phase 2 | NCT02706899 |
|  |  |  | Completed | Phase 1 | NCT01902329 |
| AGS67E | CD37 | MMAE | Completed | Phase 1 | NCT02175433 |
|  |  |  | Terminated | Phase 1 | NCT02610062 |
| STI-6129 | CD38 | Duostatin 5.2 | Recruiting | Phase 1\|Phase 2 | NCT05308225 |
| FOR46 | CD46 | MMAF | Recruiting | Phase 1\|Phase 2 | NCT05011188 |
|  |  |  | Recruiting | Phase 1 | NCT03575819 |
|  |  |  | Active, not recruiting | Phase 1 | NCT03650491 |
| SGN-CD48A | CD48 | MMAE | Terminated | Phase 1 | NCT03379584 |
| Lorvotuzumab Mertansine | CD56 | DM1 | Completed | Phase 2 | NCT02452554 |
| AMG 172 | CD70 | DM1 | Completed | Phase 1 | NCT01497821 |
| SGN-CD70A | CD70 | PBD | Completed | Phase 1 | NCT02216890 |
| Vorsetuzumab mafodotin | CD70 | MMAF | Terminated | Phase 1 | NCT01677390 |
|  |  |  | Completed | Phase 1 | NCT01015911 |
| INA03 | CD71 | MMAE | Unknown status | Early Phase 1 | NCT03957915 |
| CX-2029 | CD71 | MMAE | Recruiting | Phase 1\|Phase 2 | NCT03543813 |
| Milatuzumab-doxorubicin | CD74 | Doxorubicin | Terminated | Phase 1\|Phase 2 | NCT01101594 |
| STRO-001 | CD74 | Maytansoid | Recruiting | Phase 1 | NCT03424603 |
| Polatuzumab Vedotin | CD79b | MMAE | Recruiting | Phase 2 | NCT04479267 |
|  |  |  | Recruiting | Phase 2 | NCT05169658 |
|  |  |  | Recruiting | Phase 2 | NCT04659044 |
|  |  |  | Recruiting | Phase 2 | NCT04665765 |
|  |  |  | Suspended | Phase 1 | NCT04231877 |
| Labetuzumab govitecan | CEACAM5 | SN38 | Withdrawn | Phase 2 | NCT01915472 |
|  |  |  | Withdrawn | Phase 1\|Phase 2 | NCT01605318 |
|  |  |  | Completed | Phase 1 | NCT01270698 |
| CPO102 | Claudin 18.2 | MMAE | Not yet recruiting | Phase 1 | NCT05043987 |
| RC118-ADC | Claudin 18.2 | MMAE | Recruiting | Phase 1\|Phase 2 | NCT05205850 |
| RC108 | cMET | Microtube inhibitor | Recruiting | Phase 1 | NCT04617314 |
| SHR-A1403 | cMET | SHR152852 | Unknown status | Phase 1 | NCT03856541 |
| Telisotuzumab vedotin | cMET | MMAE | Recruiting | Phase 1 | NCT02099058 |
|  |  |  | Completed | Phase 2 | NCT03574753 |
| TR1801-ADC | cMET | PBD | Active, not recruiting | Phase 1 | NCT03859752 |
| Rovalpituzumab tesirine | DLL3 | PBD | Completed | Phase 1\|Phase 2 | NCT01901653 |
|  |  |  | Completed | Phase 2 | NCT02674568 |
|  |  |  | Terminated | Phase 1 | NCT02819999 |
|  |  |  | Completed | Phase 1 | NCT02874664 |
| SC-002 | DLL3 | PBD | Terminated | Phase 1 | NCT02500914 |
| SC-003 | DPEP3 | PBD | Terminated | Phase 1 | NCT02539719 |
| PF-06647263 | EFNA4 | Calicheamicin | Terminated | Phase 1 | NCT02078752 |
| Depatuxizumab mafodotin | EGFR | MMAF | Completed | Phase 2 | NCT02343406 |
|  |  |  | Active, not recruiting | Phase 2\|Phase 3 | NCT02573324 |
|  |  |  | Terminated | Phase 3 | NCT03419403 |
| Laprituximab emtansine | EGFR | DM1 | Terminated | Phase 1 | NCT01963715 |
| MRG003 | EGFR | MMAE | Completed | Phase 1 | NCT04868344 |
|  |  |  | Recruiting | Phase 2 | NCT04838964 |
|  |  |  | Recruiting | Phase 2 | NCT05188209 |
|  |  |  | Recruiting | Phase 2 | NCT04838548 |
|  |  |  | Recruiting | Phase 2 | NCT04868162 |
|  |  |  | Recruiting | Phase 2 | NCT05126719 |
| M1231 | EGFR/MUC1 | Hemiasterlin-related | Recruiting | Phase 1 | NCT04695847 |
| AMG 595 | EGFRvIII | DM1 | Completed | Phase 1 | NCT01475006 |
| AGS-16C3F | ENPP3 | MMAF | Completed | Phase 1 | NCT01672775 |
|  |  |  | Completed | Phase 2 | NCT02639182 |
| A166 | ERBB2 | Duostatin-5 | Active, not recruiting | Phase 1\|Phase 2 | NCT03602079 |
|  |  |  | Recruiting | Phase 1 | NCT05311397 |
| ALT-P7 | ERBB2 | MMAE | Completed | Phase 1 | NCT03281824 |
| ARX788 | ERBB2 | Amberstatin269 | Not yet recruiting | Phase 2 | NCT04983121 |
|  |  |  | Recruiting | Phase 2 | NCT04829604 |
|  |  |  | Recruiting | Phase 1 | NCT03255070 |
|  |  |  | Terminated | Phase 1 | NCT02512237 |
| BAT8001 | ERBB2 | Maytansine derivative | Unknown status | Phase 1 | NCT04189211 |
| DP303c | ERBB2 | - | Unknown status | Phase 1 | NCT04146610 |
| GQ1001 | ERBB2 | DM1 | Recruiting | Phase 1 | NCT04450732 |
| MRG002 | ERBB2 | MMAE | Not yet recruiting | Phase 2 | NCT05263869 |
|  |  |  | Recruiting | Phase 2 | NCT04924699 |
|  |  |  | Recruiting | Phase 2 | NCT04742153 |
|  |  |  | Recruiting | Phase 2 | NCT05141747 |
|  |  |  | Recruiting | Phase 2 | NCT04837508 |
|  |  |  | Not yet recruiting | Phase 2 | NCT05141786 |
|  |  |  | Recruiting | Phase 2 | NCT04839510 |
|  |  |  | Recruiting | Phase 1 | NCT04941339 |
|  |  |  | Recruiting | Phase 1\|Phase 2 | NCT04492488 |
|  |  |  | Not yet recruiting | Phase 1\|Phase 2 | NCT05338957 |
| RC48-ADC | ERBB2 | MMAE | Not yet recruiting | Phase 2 | NCT05115500 |
|  |  |  | Unknown status | Phase 1\|Phase 2 | NCT04264936 |
|  |  |  | Recruiting | Phase 2 | NCT04329429 |
|  |  |  | Active, not recruiting | Phase 2 | NCT04073602 |
| SBT6050 | ERBB2 | TLR8 agonist | Recruiting | Phase 1 | NCT04460456 |
| Trastuzumab deruxtecan | ERBB2 | DXd | Active, not recruiting | Phase 2 | NCT04132960 |
|  |  |  | Active, not recruiting | Phase 3 | NCT03734029 |
|  |  |  | Active, not recruiting | Phase 3 | NCT03523585 |
|  |  |  | Active, not recruiting | Phase 2 | NCT03248492 |
|  |  |  | Active, not recruiting | Phase 3 | NCT03529110 |
|  |  |  | Active, not recruiting | Phase 2 | NCT03505710 |
|  |  |  | Recruiting | Phase 1 | NCT04042701 |
|  |  |  | Active, not recruiting | Phase 1 | NCT03523572 |
|  |  |  | Active, not recruiting | Phase 1 | NCT03368196 |
|  |  |  | Recruiting | Phase 1 | NCT04585958 |
|  |  |  | Completed | Phase 2 | NCT03384940 |
|  |  |  | Completed | Phase 1 | NCT03366428 |
|  |  |  | Active, not recruiting | Phase 1 | NCT02564900 |
|  |  |  | Recruiting | Phase 1\|Phase 2 | NCT04644068 |
|  |  |  | Active, not recruiting | Phase 1 | NCT03383692 |
|  |  |  | Recruiting | Phase 1 | NCT04556773 |
|  |  |  | Recruiting | Phase 1\|Phase 2 | NCT04538742 |
|  |  |  | Recruiting | Phase 3 | NCT04494425 |
| Trastuzumab Duocarmazine | ERBB2 | Duocarmycin | Completed | Phase 1 | NCT02277717 |
|  |  |  | Active, not recruiting | Phase 1 | NCT04235101 |
|  |  |  | Active, not recruiting | Phase 3 | NCT03262935 |
|  |  |  | Recruiting | Phase 1 | NCT04602117 |
|  |  |  | Recruiting | Phase 2 | NCT04205630 |
|  |  |  | Recruiting | Phase 1\|Phase 2 | NCT04983238 |
| Trastuzumab emtansine | ERBB2 | DM1 | Withdrawn | Phase 2 | NCT02725541 |
|  |  |  | Unknown status | Phase 2 | NCT03225937 |
|  |  |  | Active, not recruiting | Phase 2 | NCT03587740 |
|  |  |  | Completed | Phase 1 | NCT02073916 |
|  |  |  | Recruiting | Phase 2 | NCT02448420 |
|  |  |  | Completed | Phase 1 | NCT02038010 |
|  |  |  | Completed | Phase 2 | NCT02073487 |
|  |  |  | Withdrawn | Phase 2 | NCT04351230 |
|  |  |  | Completed | Phase 1 | NCT01816035 |
|  |  |  | Active, not recruiting | Phase 2 | NCT04439110 |
|  |  |  | Active, not recruiting | Phase 1 | NCT03364348 |
|  |  |  | Recruiting | Phase 2 | NCT04197687 |
|  |  |  | Recruiting | Phase 2 | NCT04266249 |
|  |  |  | Recruiting | Phase 2 | NCT02465060 |
|  |  |  | Not yet recruiting | Phase 2 | NCT05238831 |
|  |  |  | Recruiting | Phase 1 | NCT03878524 |
| XMT-1522 | ERBB2 | MMAF-HPA | Completed | Phase 1 | NCT02952729 |
| BDC-1001 | ERBB2 | TLR8 agonist | Recruiting | Phase 1\| Phase 2 | NCT04278144 |
| SBT6050 | ERBB2 | TLR8 agonist | Active, not recruiting | Phase 1 | NCT04460456 |
| NJH395 | ERBB2 | TLR7/8 agonist | Completed | Phase 1 | NCT03696771 |
| Patritumab Deruxtecan | ERBB3 | DXd | Recruiting | Early Phase 1 | NCT04610528 |
|  |  |  | Recruiting | Phase 2 | NCT04965766 |
|  |  |  | Recruiting | Phase 2 | NCT04699630 |
|  |  |  | Active, not recruiting | Phase 1\|Phase 2 | NCT02980341 |
| Aprutumab ixadotin | FGFR2 | Auristatin W derivative | Terminated | Phase 1 | NCT02368951 |
| LY3076226 | FGFR3 | DM4 | Completed | Phase 1 | NCT02529553 |
| ASP1235 | FLT3 | AGL-0182-30 | Terminated | Phase 1 | NCT02864290 |
| Luveltamab Tazevibulin | FRα | Tubulin-targeting 3-aminophenyl hemiasterlin (SC209) | Recruiting | Phase 1 | NCT05200364 |
|  |  |  | Recruiting | Phase 1 | NCT03748186 |
| Mirvetuximab Soravtansine | FRα | DM4 | Active, not recruiting | Phase 3 | NCT04296890 |
|  |  |  | Recruiting | Phase 2 | NCT03835819 |
|  |  |  | Recruiting | Phase 2 | NCT04606914 |
|  |  |  | Recruiting | Phase 3 | NCT04209855 |
|  |  |  | Completed | Phase 1\|Phase 2 | NCT02606305 |
|  |  |  | Recruiting | Phase 2 | NCT05041257 |
|  |  |  | Completed | Phase 3 | NCT02631876 |
| MORAb-202 | FRα | Eribulin | Active, not recruiting | Phase 1\|Phase 2 | NCT04300556 |
| MLN0264 | GCC | MMAE | Completed | Phase 1 | NCT01577758 |
| OBI-999 | GH | MMAE | Recruiting | Phase 1\|Phase 2 | NCT04084366 |
| Glembatumumab Vedotin | GPNMB | MMAE | Withdrawn | Early Phase 1 | NCT03473691 |
|  |  |  | Completed | Phase 2 | NCT01997333 |
|  |  |  | Withdrawn | Phase 1\|Phase 2 | NCT03326258 |
|  |  |  | Completed | Phase 2 | NCT02363283 |
|  |  |  | Terminated | Phase 2 | NCT02302339 |
|  |  |  | Completed | Phase 2 | NCT02487979 |
| TAK-164 | GUCY2C (extracellular domain of guanylyl cyclase C) | DGN549 | Terminated | Phase 1 | NCT03449030 |
| Lonigutamab Ugodotin | IGF-1R | Ugodotin | Recruiting | Phase 1\|Phase 2 | NCT03316638 |
| SGN-15 | Lewis Y antigen | Doxorubicin | Terminated | Phase 2 | NCT00051584 |
|  |  |  | Completed | Phase 2 | NCT00031187 |
| Ladiratuzumab vedotin | LIV-1 | MMAE | Recruiting | Phase 1 | NCT01969643 |
| ABBV-085 | LRRC15 | MMAE | Completed | Phase 1 | NCT02565758 |
| Lupartumab Amadotin | LYPD3 | Auristatin W derivative | Terminated | Phase 1 | NCT02134197 |
| Anetumab ravtansine | Mesothelin | DM4 | Completed | Phase 1 | NCT01439152 |
|  |  |  | Completed | Phase 2 | NCT03023722 |
|  |  |  | Terminated | Phase 1 | NCT03455556 |
|  |  |  | Terminated | Phase 2 | NCT02839681 |
|  |  |  | Completed | Phase 1 | NCT02696642 |
|  |  |  | Completed | Phase 1 | NCT02751918 |
|  |  |  | Completed | Phase 1 | NCT02485119 |
| BMS-986148 | Mesothelin | Tubulysin | Terminated | Phase 1\|Phase 2 | NCT02341625 |
| RC88 | Mesothelin | MMAE | Recruiting | Phase 1 | NCT04175847 |
| BYON3521 | MET | Duocarmycin hydroxybenzamide azaindole (DUBA) | Recruiting | Phase 1 | NCT05323045 |
| REGN5093-M114 | METxMET | Maytansinoid | Recruiting | Phase 1\|Phase 2 | NCT04982224 |
| Upifitamab Rilsodotin | NaPi2b | Auristatin DolaLock | Recruiting | Phase 1\|Phase 2 | NCT04907968 |
|  |  |  | Recruiting | Phase 1\|Phase 2 | NCT03319628 |
|  |  |  | Not yet recruiting | Phase 3 | NCT05329545 |
| XMT-1592 | NaPi2b | Auristatin DolaLock | Recruiting | Phase 1\|Phase 2 | NCT04396340 |
| 9MW2821 | Nectin 4 | MMAE | Not yet recruiting | Phase 1\|Phase 2 | NCT05216965 |
| Enfortumab vedotin | Nectin 4 | MMAE | Active, not recruiting | Phase 2 | NCT04995419 |
|  |  |  | Recruiting | Phase 1 | NCT04963153 |
|  |  |  | Active, not recruiting | Phase 3 | NCT03474107 |
|  |  |  | Active, not recruiting | Phase 1 | NCT02091999 |
|  |  |  | Recruiting | Phase 1\|Phase 2 | NCT03288545 |
|  |  |  | Active, not recruiting | Phase 2 | NCT03219333 |
|  |  |  | Recruiting | Phase 2\|Phase 3 | NCT04887870 |
|  |  |  | Completed | Phase 1 | NCT03070990 |
|  |  |  | Recruiting | Phase 2 | NCT03606174 |
|  |  |  | Recruiting | Phase 1 | NCT04878029 |
|  |  |  | Recruiting | Phase 1 | NCT04724018 |
| ARX517 | PSMA | Amberstatin269 | Recruiting | Phase 1 | NCT04662580 |
| PSMA ADC | PSMA | MMAE | Completed | Phase 1 | NCT01414283 |
|  |  |  | Completed | Phase 1 | NCT01414296 |
|  |  |  | Completed | Phase 2 | NCT01695044 |
|  |  |  | Completed | Phase 2 | NCT02020135 |
| Cofetuzumab Pelidotin | PTK7 | Aur0101 | Recruiting | Phase 1 | NCT04189614 |
| CS5001 | ROR1 | PBD | Not yet recruiting | Phase 1 | NCT05279300 |
| NBE-002 | ROR1 | PNU-159682 | Recruiting | Phase 1\|Phase 2 | NCT04441099 |
| Ozuriftamab Vedotin | ROR2 | MMAE | Recruiting | Phase 1\|Phase 2 | NCT03504488 |
|  |  |  | Not yet recruiting | Phase 2 | NCT05271604 |
| Azintuxizumab Vedotin | SLAMF7 | MMAE | Terminated | Phase 1 | NCT02462525 |
| ASG-5ME | SLC44A4 | MMAE | Completed | Phase 1 | NCT01166490 |
| AGS15E | SLITRK6 | MMAE | Completed | Phase 1 | NCT01963052 |
| HKT288 | TAA | DM4 | Terminated | Phase 1 | NCT02947152 |
| TORL-1-23 | TAA | - | Recruiting | Phase 1 | NCT05103683 |
| TORL-2-307-ADC | TAA | - | Recruiting | Phase 1 | NCT05156866 |
| MRG004A | TF | - | Recruiting | Phase 1\|Phase 2 | NCT04843709 |
| Tisotumab vedotin | TF | MMAE | Completed | Phase 1\|Phase 2 | NCT02552121 |
|  |  |  | Completed | Phase 1\|Phase 2 | NCT02001623 |
|  |  |  | Completed | Phase 2 | NCT03657043 |
|  |  |  | Active, not recruiting | Phase 2 | NCT03438396 |
| CDX-014 | TIM-1 | MMAE | Terminated | Phase 1 | NCT02837991 |
| BIO-106 | TROP2 | - | Recruiting | Phase 1\|Phase 2 | NCT05320588 |
| Datopotamab deruxtecan | TROP2 | DXd | Recruiting | Phase 2 | NCT04940325 |
|  |  |  | Recruiting | Phase 3 | NCT05104866 |
| FDA018-ADC | TROP2 | - | Recruiting | Phase 1 | NCT05174637 |
| Sacituzumab Govitecan | TROP2 | Topoisomerase-1 (TOP1) inhibitor | Recruiting | Phase 1\|Phase 2 | NCT04039230 |
|  |  |  | Recruiting | Phase 3 | NCT04595565 |
|  |  |  | Completed | Phase 1\|Phase 2 | NCT01631552 |
|  |  |  | Recruiting | Phase 2 | NCT03725761 |
|  |  |  | Recruiting | Phase 2 | NCT04434040 |
|  |  |  | Enrolling by invitation | Phase 4 | NCT04319198 |
|  |  |  | Withdrawn | Phase 2 | NCT02161679 |
|  |  |  | Recruiting | Phase 2 | NCT04647916 |
|  |  |  | Recruiting | Phase 1 | NCT04617522 |
|  |  |  | Recruiting | Phase 2 | NCT04251416 |
|  |  |  | Recruiting | Phase 1\|Phase 2 | NCT04826341 |
|  |  |  | Not yet recruiting | Phase 1 | NCT05143229 |
|  |  |  | Recruiting | Phase 3 | NCT04639986 |
|  |  |  | Recruiting | Phase 1 | NCT04724018 |
| SKB264 | TROP2 | Belotecan-derived payload | Recruiting | Phase 1\|Phase 2 | NCT04152499 |
| STI-3258 | TROP2 | - | Not yet recruiting | Phase 1 | NCT05060276 |

ADC FDA approved

ADC selective payload

ADC bispecific

PROBODY
