# Supplementary material for: Uncovering therapeutic opportunities in the clinical development of antibody‐drug conjugates
Source: Clin Transl Med. 2023 Sep 22;13(9):e1329. doi: 10.1002/ctm2.1329 (PMC10517221; doi:10.1002/ctm2.1329)
Supplement: Supplementary file 3 — Table S2 Efficacy of approved ADCs. [file CTM2-13-e1329-s001.docx]

| ADC | FDA Indication | Study | Design | Primary Objective | Results |
| --- | --- | --- | --- | --- | --- |
| Mirvetuximab soravtansine | Ovarian cancer platinum resistant | Study 0417 (SORAYA)  NCT04296890 | Phase 3 study | ORR | ORR 31.7%, DOR 6.9 m. |
| Tisotumab vedotin | Cervical cancer | InnovaTV 204  NCT03438396 | Phase 2 | ORR and DOR | ORR 24%, DOR 8.3 m. |
| Loncastuximab tesirine | B cell Lymphoma | LOTIS-2. NCT03589469 | Phase 2 | ORR | ORR 48.3%, CR 24.1% and DOR 10.3 m. |
| Sacituzumab govitecan | Triple negative metastatic breast cancer | ASCENT. NCT02574455 | Phase 3 | PFS | PFS 5.6 vs 1.7m.  OS 12.1 vs 6.7m.  ORR 35% vs 5%. |
| Sacituzumab govitecan | Metastatic Breast Cancer RH+/HER2- | TROPiCS-02. NCT03901339 | Phase 3 | PFS | PFS 5.5 vs 4m (HR 0.66). |
| Trastuzumab deruxtecan | Metastatic Breast Cancer HER2+ | DESTINY-Breast03.  NCT03529110 | Phase 3 | PFS | PFS 28.8 vs 6.8.  OS NR con HR 0.64.  ORR 79.7 vs 34.2%. |
| Trastuzumab deruxtecan | Metastatic Breast Cancer HER2-low | DESTINY-Breast04.  NCT03734029 | Phase 3 | PFS in RH+ | PFS 10.1 vs 5.4m  OS 23.9 17.5m |
| Trastuzumab deruxtecan | Metastatic NSCLC | DESTINY-Lung02 | Phase 2 | ORR | ORR 58%, DOR 8.7 m. |
| Trastuzumab deruxtecan | Metastatic Gastric | DESTINY-Gastric01.  NCT03329690 | Phase 2 | OS, ORR | OS 12.5 vs 8,4. HR 0.59.  ORR 40.5% vs 11.3%. |
| Enfortumab vedotina | Metastatic Urothelial | EV-301.  NCT03474107 | Phase 3 | OS | OS 12.88 vs 8.97m. HR 0.70.  PFS 5.55 vs 3.71m. HR 0.62. |
| Polatuzumab vedotin | B cell Lymphoma | GO29365.  NCT02257567 | Phase 2 | CR, DOR | CR 40% vs 17.5%.  DOR 9.5 vs 3.7m. |
| Inotuzumab ozogamicin | LLA CD22+ | INO-VATE ALL NCT01564784 | Phase 3 | CR, OS | CR 80.7% vs 29.4%.  DOR 4.6 vs 3.1m.  PFS 5 vs 1.8m  OS 7.7 vs 6.7m |
| Trastuzumab emtansine | Metastatic Breast Cancer HER2+ | EMILIA NCT00829166 | Phase 3 | PFS, OS | PFS 9.6m vs 6.4 m.  OS 30.9 vs 25.1m. |
| Trastuzumab emtansine | Metastatic Breast Cancer HER2+ | KATHERINE NCT01772472 | Phase 3 | iDFS | iDFS at 3 years: 88.3% vs 77%, HR 0.50. |
| Brentuximab vedotin | Hodking Lymphoma stage III or IV | ECHELON-1  NCT01712490 | Phase 3 | Modified PFS | mPFS not reached, HR 0,77 y p 0,035. |
| Gemtuzumab ozogamicina in combination | LMA CD33+ | ALFA-0701 NCT00927498 | Phase 3 | EFS | EFS at 2 years: 17,1% vs 40,8%. HR 0,58. |
| Gemtuzumab ozogamicina in monotherapy | LMA CD33+ | AML-19  NCT00091234 | Phase 3 | OS | OS 4,9 vs 3,6m. HR 0,69. |

**Supplementary table 2**
