# Supplementary material for: Uncovering therapeutic opportunities in the clinical development of antibody‐drug conjugates
Source: Clin Transl Med. 2023 Sep 22;13(9):e1329. doi: 10.1002/ctm2.1329 (PMC10517221; doi:10.1002/ctm2.1329)
Supplement: Supplementary file 5 — Table S4 Number of samples for each tumour and normal tissue that have been genomically analyzed (TCGA and GTEx) [file CTM2-13-e1329-s002.docx]

**GEPIA2**

| TCGA | Detail | Tumor | Normal | GTEx (Normal tissue) | Number of samples |
| --- | --- | --- | --- | --- | --- |
| ACC | Adrenocortical carcinoma | 77 | - | Adrenal Gland | 128 |
| BLCA | Bladder Urothelial Carcinoma | 404 | 19 | Bladder | 9 |
| BRCA | Breast invasive carcinoma | 1085 | 112 | Breast | 179 |
| CESC | Cervical squamous cell carcinoma and endocervical adenocarcinoma | 306 | 3 | Cervix Uteri | 10 |
| CHOL | Cholangio carcinoma | 36 | 9 | - | - |
| COAD | Colon adenocarcinoma | 275 | 41 | Colon | 308 |
| DLBC | Lymphoid Neoplasm Diffuse Large B-cell Lymphoma | 47 | - | Blood | 337 |
| ESCA | Esophageal carcinoma | 182 | 13 | Esophagus | 273 |
| GBM | Glioblastoma multiforme | 163 | - | Brain | 207 |
| HNSC | Head and Neck squamous cell carcinoma | 519 | 44 | - | - |
| KICH | Kidney Chromophobe | 66 | 25 | Kidney | 28 |
| KIRC | Kidney renal clear cell carcinoma | 523 | 72 | Kidney | 28 |
| KIRP | Kidney renal papillary cell carcinoma | 286 | 32 | Kidney | 28 |
| LAML | Acute Myeloid Leukemia | 173 | - | Bone Marrow | 70 |
| LGG | Brain Lower Grade Glioma | 518 | - | Brain | 207 |
| LIHC | Liver hepatocellular carcinoma | 369 | 50 | Liver | 110 |
| LUAD | Lung adenocarcinoma | 483 | 59 | Lung | 288 |
| LUSC | Lung squamous cell carcinoma | 486 | 50 | Lung | 288 |
| MESO | Mesothelioma | 87 | - | - | - |
| OV | Ovarian serous cystadenocarcinoma | 426 | - | Ovary | 88 |
| PAAD | Pancreatic adenocarcinoma | 179 | 4 | Pancreas | 167 |
| PCPG | Pheochromocytoma and Paraganglioma | 182 | 3 | - | - |
| PRAD | Prostate adenocarcinoma | 492 | 52 | Prostate | 100 |
| READ | Rectum adenocarcinoma | 92 | 10 | Colon | 308 |
| SARC | Sarcoma | 262 | 2 | - | - |
| SKCM | Skin Cutaneous Melanoma | 461 | 1 | Skin | 557 |
| STAD | Stomach adenocarcinoma | 408 | 36 | Stomach | 175 |
| TGCT | Testicular Germ Cell Tumors | 137 | - | Testis | 165 |
| THCA | Thyroid carcinoma | 512 | 59 | Thyroid | 278 |
| THYM | Thymoma | 118 | 2 | Blood | 337 |
| UCEC | Uterine Corpus Endometrial Carcinoma | 174 | 13 | Uterus | 78 |
| UCS | Uterine Carcinosarcoma | 57 | - | Uterus | 78 |
| UVM | Uveal Melanoma | 79 | - | - | - |
|  |  |  |  | Adipose Tissue | 515 |
|  |  |  |  | Lymphocytes | 107 |
|  |  |  |  | Blood Vessel | 606 |
|  |  |  |  | Brain | 945 |
|  |  |  |  | Esophagus | 382 |
|  |  |  |  | Fallopian Tube | 5 |
|  |  |  |  | Heart | 377 |
|  |  |  |  | Muscle | 396 |
|  |  |  |  | Nerve | 278 |
|  |  |  |  | Pituitary | 107 |
|  |  |  |  | Salivary Gland | 55 |
|  |  |  |  | Fibroblasts | 256 |
|  |  |  |  | Small Intestine | 92 |
|  |  |  |  | Spleen | 100 |
|  |  |  |  | Vagina | 85 |

**GENT2**

| **Subtype** | **Number of samples** |
| --- | --- |
| HER2 | 230 |
| TNBC | 251 |
| Luminal | 640 |
| Leukemia | 3254 |
| Lymphoma | 1413 |
| Myeloma | 343 |

**Supplementary table 4**
